# Exploration of the costs of accessing health services: data from a longitudinal study of young people in transition from paediatric to adult services

**DOI:** 10.1186/s12913-021-06280-z

**Published:** 2021-03-21

**Authors:** Julija Simpson, Tomos Robinson, Luke Vale

**Affiliations:** Health Economics Group, Population Health Sciences Institute, Newcastle University, Newcastle upon Tyne, UK

**Keywords:** Transition care, Health economics, Economic evaluation, Patient costs

## Abstract

**Background:**

Economic evaluations that include the patient perspective often base their estimates of patient time and travel costs on data collected at a single point in time. This, however, may be inaccurate if the costs of accessing care change substantially over time, as may be the case for young people in transition from paediatric to adult health services.

**Aims:**

The aim of this study was to explore the differences in these time and travel costs between two data collection points for young individuals in transition between health care services, and thus to provide an insight of whether such costs should be collected more than once.

**Methods:**

Descriptive statistics and regression modelling were used to estimate the average difference in costs between the two points of data collection, as well as the potential drivers of those cost differences.

**Results:**

We found a small difference in costs between the two time points, equal to -£45.78 [95% CI: − 89.70 to − 1.86]. The results were largely driven by changes in the unit cost of visits and in the number of attendances.

**Conclusions:**

A simple and common assumption that patient costs could be collected at a single time point cannot be made in the context of our study. When deciding on the frequency of elicitation of patient costs, future studies should consider the relative impacts of additional data collection on the estimates of efficiency, inequalities and resource implications for collecting new data.

**Supplementary Information:**

The online version contains supplementary material available at 10.1186/s12913-021-06280-z.

## Introduction

Economic evaluations can take a variety of different perspectives about whose costs and benefits are important to consider. In England, the reference case perspective recommended by NICE (National Institute for Care and Excellence) is the National Health Service and Personal and Social Services (NHS & PSS) [1], with a public sector perspective adopted if public health components are considered [2]. However, in other countries a different view is taken. For example, in Germany the independent Institute for Quality and Efficiency in Health Care (Institut für Qualität und Wirtschaftlichkeit im Gesundheitswesen, IQWiG) takes the perspective of the statutory health insurance. With this perspective, costs borne by those covered by the insurance should also be considered. This could mean co-payments for care but also the costs of accessing care [3]. As the approaches adopted by NICE and IQWiG (and other health technology assessment agencies worldwide) illustrate, a key requirement for any health technology assessment is that studies including an economic evaluation state the perspective from which costs and benefits are considered and how these will be collected [4].

Whilst practice between countries varies, there is also variation in practice within countries. In a review of 95 studies funded by the UK National Institute for Health Research (NIHR) Health Technology Assessment (HTA) programme published in June 2009, 26 studies (27%) included a perspective that accounted for ‘patient’ costs [5]. Patient costs typically were out-of-pocket costs for the purchase or access to care that are borne by patients and their families, friends and/or unpaid carers.

A further element of patient costs that could be considered is the time and travel costs incurred when accessing care. These costs may be incurred both when accessing purchased health care but also where care is free at the point of use, as it is for much care in the UK and in many other European countries [6, 7]. Consideration of these costs is important for two reasons. First, an intervention that is judged cost-effective from a narrower perspective, e.g. NHS & PSS within the UK, might not be when a wider perspective is taken. Second, the transfer of costs may have an impact on health and other inequalities. This is especially important, given that those that need to access care more are already more likely to be in socio-economically deprived groups, and have the least ability to bear these costs.

When conducting a prospective economic evaluation, it is possible to elicit total time and travel costs for each study participant. These total costs will be driven by the frequency of service use and also the time and travel cost of each episode of service use. The frequency of service use is often collected as part of studies either via a participant completed questionnaires, case report forms or routinely collected data. Data on the time and travel cost of each episode of care can be elicited direct from study participants but this can be burdensome both for study teams and study participants to collect. How frequently such data should be collected during a study follow-up period is unclear. It might be the case that the time and travel costs of each episode of care do not change substantially over time (for example, the unit patient cost of attending an outpatient appointment is assumed constant over time). If such data could be collected once, this would reduce respondent burden. This would not be appropriate if the costs of accessing care change substantially over time. This latter situation would occur if the level of dependency of patients and families changes over time.

How frequent data collection of time and travel costs should be was explored as a part of the NIHR (National Institute for Health Research) funded Transition Programme. This programme of work was used because it focused on the movement of young adults with chronic health needs as they moved from child to adult services. All the participants had one of three chronic conditions (autism spectrum disorder with an additional mental health need, cerebral palsy or diabetes). For many of the young people, how and how much health care they accessed changed over time as their level of independence changed. The three conditions were chosen as chronic illnesses as they were expected to exhibit different patterns of care. For example, diabetes is prone to deteriorate during the adolescent years, although there are well organised child and adult services [8]. Cerebral palsy is associated with symptoms that interfere with daily living such as pain, spasticity or seizures. Management of cerebral palsy requires multidisciplinary co-ordination, provided in childhood but rarely in adulthood [9]. Likewise, whilst services are available during childhood for those with autism spectrum disorder, adult services are rarely available [10]. Thus, whilst the frequency of use of services may vary over time, it is possible that the costs of accessing care will differ as well. The aim of this study was to explore the differences in these time and travel costs between the data collection points, and thus to provide an insight of whether such costs should be collected more than once.

## Methods

### Brief description of case study

At any given time in the UK, approximately 156,000 young people undergo transition from paediatric to adult services (i.e. 700 per NHS trust × 223 trusts in the UK) [8, 11]. The Transition Research Programme sought to establish how successful transition; defined as “the purposeful, planned process that addresses the medical, psychosocial, educational, and vocational needs of adolescents and young adults with chronic medical and physical conditions as they move from child-centred to adult-oriented health care systems” ( [8] , p.1) - can be facilitated to improve health and social outcomes.

One component of this work was a 3 year longitudinal data cohort study on young people (*n* = 374) from England and Northern Ireland with one of the three long term conditions described above as they experienced transition.

As service delivery could not be assumed to remain constant over time for this study population, time and travel costs were collected at two time points. Specifically, young people (YPs) completed Time and Travel Questionnaires via structured interview with a researcher at both visit two (approximately 1 year after entry to the study baseline) and visit three (approximately 1 year after visit two). In circumstances where it had not been possible to conduct visit three, data collection was attempted again at the final visit (visit four), which took place the following (and final) study year. To simplify the terminology, we will refer to visit 2 as ‘time point one’ and visits 3 or 4 as ‘time point two’ from this point onwards.

### Data

Data were collected on type of health service accessed, distance travelled, method of transport, any fares or parking changes; time taken both travelling and at the health care venue. These data were collected both for the young person and for any accompanying person/people. For both the young person and any accompanying person/people information was sought on what activities were displaced accessing the health care. Additionally, the total number of visits to each type of health care provider in that year was collected as was when the YP had transferred to adult services. We also included a measure of deprivation for each participant. Specifically, the sample was divided into five categories (quintiles) based on their index of multiple deprivation (IMD), calculated from the given post code data, with one and five being the categories of most and least deprived respectively. It should be noted that the indices of deprivation are calculated separately for each of the four devolved nations in the UK. We therefore obtained deprivation quintiles for both England [12] and Northern Ireland [13]. Both of these measures consist of seven domains, including Income, Employment, Health, Education, Skills and Training, Access to Housing Services, Living Environment; and Crime & Disorder [12, 13].

For each participant, travel costs and time costs were calculated from these averages collected for i) the entire sample across both data collection periods ii) each data collection point, iii) each of the three conditions. For travel costs, data on journey miles were combined with routine source information on the cost per mile [14] and parking cost information as reported by the YP, to estimate car journey costs. For public transport journeys, fare data as reported by participants was used to estimate travel costs.

Time data were collected in their natural units (e.g. hours and minutes). The cost of this time was based upon the activity displaced. For leisure time (either/both YP and/or accompanying person), we used the Department of Transport estimates [15]. For paid work we used the national median wage rate per hour [16]. For the accompanying people we used the estimate for continuous employment and for YPs we used the overall national average including both continuous and discontinuous employment. These unit time costs, measured in terms of their natural and monetary terms were then combined with estimates of number of contacts (to calculate total time costs for accessing care within a given year. All costs in the analyses were at the 2017 UK£ price level.

### Methods

STATA (version 14) [17] was used to compile summary statistics regarding venues, costs and time for Diabetes, Cerebral Palsy and Autistic Spectrum Disorder patients at two time points (where the time points were 1–2 years apart). In order to perform the analysis, the sample was split by speciality and/or transfer status. No sample size calculations were performed as this was a secondary analysis of original data.

We used regression modelling to estimate the association between the total time and travel costs (C) and a dummy variable for the time point 2 (TP), whilst controlling for a vector (X) of other characteristics also predicted to be related to time and travel cost, including speciality, the type of service (adult or child), age, gender and deprivation. The exact specification of the econometric model estimated can be given as:

$$ {C}_i={\beta}_0+{\beta}_1T{P}_i+\pi {X}_i+{u}_i $$

where subscript *i* refers to the study participant, *β*_0_ represents the constant term, *β*_1_ represents the vector coefficient associated with the dummy time point variable, *π* represents the vector coefficients associated with the controlling characteristics and u represents the individual specific idiosyncratic error term that is assumed to be normally distributed.

To take account of the non-linear distribution of the total cost variable, we estimated a pooled generalized linear model (GLM) [18] with standard errors clustered at the individual level. We used the modified Park Test [19] to determine the distribution and link function that best fitted the data. In this case, the gamma distribution and log link function fitted the data the best. As the estimated coefficients from this model cannot be directly interpreted as marginal effects, we calculated marginal effects ex post.

To investigate the potential reasons for a difference between the two time points, we also used non-parametric Wilcoxon Matched Pairs Signed-Ranks Tests [20] to investigate whether the number of visits or the cost per visit varied significantly between the two data collection points.

## Results

### Sample characteristics

The number of patients who had complete data for both time points, and thus included in our analysis was 221 (57% male). There was little evidence that those that only completed the questionnaire once were different from those who had completed the questionnaire at both time points. For example, both samples had similar male to female ratios and were similarly distributed across the three conditions. Participants were also similar with regard to transfer status at both time points (see Supplementary file 1).

In terms of the three conditions, 45 % of participants had diabetes, 30% had ASD and 25% had Cerebral Palsy. Around a third (29%) of all participants had already transferred to adult services by Time Point 1, and further 27% had transferred by Time Point 2. Thus, just over a half of participants (56%) had transferred by the end of the study. The key sample characteristics are illustrated in Table 1. Some of the key visit-level characteristics, such as types of healthcare services accessed across the three conditions, are available in Supplementary file 2.

**Table 1:** Sample Characteristics

Sample characteristic	Mean (SD)
N	221
Age	20.04 (1.30)
%F	43
%Diabetes	45
%CP	25
%ASD	30
%Transferred at T1	29
%Transferred at T2	56
Total Cost at Time Point 1	246.73 (407.39)
Total Cost at Time Point 2	196.06 (237.05)
% IMD 1st Quintile	16
% IMD 2nd Quintile	18
% IMD 3rd Quintile	22
% IMD 4th Quintile	22
% IMD 5th Quintile	22

Mean values and standard deviations presented for continuous variables. Percentage of sample in each category presented for categorical variables

### Differences in time and travel cost per participant

Table 2 shows the marginal effects from the GLM regression models on total time and travel costs. Once the full set of controlling variables were included in the model specification, the average total cost was estimated to be -£45.78 [95% CI:-89.70 to − 1.86] per participant lower in time point 2 as compared to time point 1.

**Table 2:** Association between time collection point and total time and travel costs

Variable	Marginal Effect	95% CI
Time Point 2	−45.78** (22.41)	−89.70, − 1.86
Autism	(Reference)	
Cerebral Palsy	121.06*** (39.50)	43.63, 198.49
Diabetes	85.59*** (19.74)	46.91, 124.27
Adult Services	− 48.17* (26.15)	− 99.43, 3.08
Age	6.99 (11.89)	−16.32, 30.30
Female	−63.87*** (24.03)	− 110.97, − 16.76
IMD Quintile 1	(Reference)	
IMD Quintile 2	184.66*** (42.99)	100.40, 268.92
IMD Quintile 3	127.22*** (39.10)	50.58, 203.86
IMD Quintile 4	82.89*** (22.67)	38.46, 127.33
IMD Quintile 5	63.50** (22.88)	18.66, 108.33
Log Likelihood	− 2783.50	
N	442	

Marginal effects from GLM with a gamma distribution and a log link function

Cluster robust standard errors in parentheses. *** *p* < 0.01, ** *p* < 0.05, * *p* < 0.1.

Both cerebral palsy and diabetes were associated with higher total time and travel costs compared with autism reflecting the nature of service provision for these conditions in both child and adult services. While there was no evidence of any association with total costs with age, we found some evidence of differences in costs by level of deprivation (though no clear trend displayed across the categories) and due to having transferred to adult services (which have lower costs relative to child services).

### Decomposing the total costs per participant

As shown in Table 2, the time and travel costs were lower at time point 2 than at time point 1 by approximately £45.78. The potential reasons for this difference include different type of services accessed (i.e. adult or child - potentially as a result of transferring between services), changes in the cost of access per visit and changes in the number of visits attended.

In terms of different costs based on types of services accessed during the two time points, the total costs were lower in Adult Services – the coefficient in Table 2 is negative (− 47.17) and statistically significant (at 10% level), indicating weak evidence of a difference.

For the cost per visit (Table 3), there was evidence that the cost was lower at time point 2 compared with time point 1 (£2.70, *p* < 0.05). This decrease in cost was largely driven by the ASD patients with a statistically significant decrease of £1.93 (*p <* 0.05) per visit at time point 2.

**Table 3:** Differences in the number attendances and cost per visit

Variable	Time Point 1	Time Point 2	Mean Difference	Wilcoxon Matched Pairs Signed-Ranks Test Statistic
**ASD (*N*** **= 134)**				
Number of Attendances	6.69	5.90	−0.79	0.300
Cost per Visit	24.03	22.10	−1.93**	0.013
**Cerebral Palsy (*N*** **= 108)**				
Number of Attendances	7.24	6.17	−1.07	0.106
Cost per Visit	40.46	35.75	−4.71	0.431
**Diabetes (*N*** **= 200)**				
Number of Attendances	6.59	6.36	−0.23*	0.086
Cost per Visit	38.12	35.52	−2.60	0.291
**Overall (*N*** **= 442)**				
Number of Attendances	6.78	6.17	−0.61**	0.011
Cost per Visit	38.14	35.44	−2.70**	0.025

Wilcoxon Matched Pairs Signed-Ranks Test Statistics *** *p* < 0.01, ** *p* < 0.05, * *p* < 0.1.

As shown in Table 3, we also found evidence of a difference in the number of different visits attended between the two time points overall (0.61, *p <* 0.05), however these differences were not statistically significant in the individual conditions, likely due to the small sample sizes.

## Discussion

Measuring costs in economic evaluations can be challenging, this is especially the case when estimating costs that fall on patients and their families of using care. Such costs are potentially important in all health care systems even those such as the UK NHS where care is often free at the point of use. Arguably, failure to capture such costs may lead to the inefficient allocation of resources from a societal perspective [21]. It has however been argued by some that such costs are not relevant as the focus should be on the use of the health care provider budget [1]. Whilst such arguments are, and have been, much debated the failure to measure such costs means that the impact on inequalities is not assessed – the costs of accessing health care almost by definition fall on those in ill health and disproportionately on the poorest in society who are the least able to bear them [22].

Prospective studies offer the opportunity to elicit such cost data directly from study participants and their families. Naively, it could be assumed that very detailed data could be collected from participants on an ongoing basis. In reality, the practicalities of research intrude, as new data collection is costly and imposes a considerable burden on participants and study staff. To alleviate this burden one solution is to elicit data only at fixed time points in a study follow-up. This then raises the question how often should such data be collected? The answers to such questions depend, in part, on the nature of the study question but to investigate this we looked at the time and travel costs of accessing care change when study participants with ongoing chronic conditions were going through very large transitions in care.

The individuals in our study each were living with one chronic condition (cerebral palsy, autism spectrum disorder or diabetes) and each was in the process of transferring from paediatric to adult services. Participants provided information to estimate their costs of accessing care at two time periods and we found that the time and travel costs decreased by an average of £46 between the two follow-up periods, with the 95% Confidence Interval ranging from £-90 to -£2. We found that the difference in costs was potentially driven by the lower unit (time and travel) cost of a typical visit, lower number of attendances, followed by being in adult services. Nevertheless, although the difference in costs between the two time points was statistically significant, our study was likely to be underpowered due to low sample size available (*n* = 221), this meant that the confidence intervals surrounding the differences in costs may include differences that are not economically important. It should also be noted that, in the context of an economic evaluation, the focus is often not on statistically significant results but on the balance of probabilities with respect to cost (and effects) as assessed through either stochastic or probabilistic analyses.

In addition, an important consideration includes whether a reduction of £45.78 per participant at the second data collection point is economically meaningful. This depends on the relative balance between the costs and benefits gained from additional data collection. The benefits in this case would include gains in precision - in terms of efficiency and in terms of estimating the impacts on inequalities. The costs would include the total resource burden falling both on the study participants and the research budgets.

In the context of our study – in the clinical setting including mostly hospital visits and large numbers of people - the gains in efficiency resulting from the second data collection could be non-trivial. More specifically, the difference of £45.78 is equivalent to 33% of the cost to health services of a hospital visit (£138 on average) in the UK [23]. This could also be of particular importance in the broader context of economic evaluations of interventions that require regular monitoring and traveling [24, 25]. Also, given that the number of young people undergoing transition at any given point in time in the UK is approximately 156,000, the total societal impact of the estimated difference in costs would be substantial.

Since the time and travel costs considered in this study fall patients and families, it is also important to consider the impact on inequalities. It is well-documented that young individuals with long-term conditions experience socio-economic disadvantage relative to the general population. For example, in the UK, young adults living in more deprived areas experience type 1 Diabetes outcomes that are significantly worse compared to those from the least deprived areas [26]. Similarly, evidence shows that the risks of experiencing CP and ASD are also pronounced for those from socially disadvantaged backgrounds [27, 28]. The difference of £45.78 might therefore be a non-trivial amount for these individuals and should be considered in the context of economic evaluations involving patient costs for vulnerable populations.

## Strengths and limitations

Reliable cost data forms the foundation of economic evaluations and comprises a major part of the research budget. However, much of the literature on cost data collection for economic evaluations is focused on questions pertaining to health care utilisation, whereas little is known about the issues relating to the nature and frequency of data collection when patient costs are concerned. Our study makes a unique contribution to this limited body of evidence by providing robust estimates of time and travel costs for two time points in the context of young people experiencing transition, and provides empirical evidence of the relative merits of collecting such data more than once. To our knowledge, this is the first such study to date.

It should be noted, however, that the study is not without limitations. First, given that only 56% of our study participants had transferred to adult services by time point 2, we were limited in terms of providing explanations for the difference in costs between the two time points. However, we felt that this is a different, albeit related, question that should be explored in future research. Second, we relied on self-reported data which may not accurately represent the actual distances travelled or time spent, and may be subject to recall bias. Nevertheless, given the inherent difficulties associated with obtaining medical records [29], deriving data based on patient recall is a common and well-established way of measuring resource use in economic evaluations [30], and according to a recent review of resource use measures in UK economic evaluations, patient reports correlate well with medical records [5], thus strengthening reliability in our estimates. Further, it would be plausible to expect that any inaccuracies resulting in participant self-reports would have been present in both time periods and therefore should have not affected the overall estimate of the difference in costs. In addition, the frequency of visits for each participant was validated by the research team [8], and although we had no exact way of validating the travel time and cost estimates, our results appear to fit well with our expectations. More specifically, the majority of our regression coefficients resulted in the expected direction. For example, at time point 2, Diabetes (on average) has the highest number of visits and also the highest total cost. This mirrors the findings of the Transition study [8] which found that adult services for Diabetes are better organised than those for ASD patients, for whom some of the adult’s services are not routinely provided. Also, the Transition study found that, across the study period, the satisfaction with visits worsened for those with ASD and CP (but remained stable for Diabetes). This could be associated with the lower number of attendances at time point 2 for ASD and CP, relative to Diabetes. Moreover, the unit costs of time and travel per health care visit (equal to approximately £38) in our study are similar to the average costs per health care visit for in the UK for other conditions. For instance the unit cost per visit is £24 for secondary care anticoagulation clinic [31] and £28 for colorectal cancer screening [32] (in 2017 prices), which provides further confidence in our results. Finally, as expected, we found that the number of health service contacts fell between the two time points across all three conditions – a finding that has been well-documented in the literature on Transition [33], thus reiterating the need for future research in this area.

## Conclusion

Our study aimed to assessed whether it is worthwhile to collect participant time and travel cost data more than once in the context where young people transition between paediatric and adult services. We found that a simple and common assumption that one data collection is enough cannot be made in this case. When deciding on the frequency of elicitation of patient costs, future studies should consider the relative impacts of additional data collection on the estimates of efficiency, inequalities and resource implications for collecting new data.

## Supplementary Information

**Additional file 1.**

**Additional file 2.**

## Data Availability

The data that support the findings of this study are available from Newcastle University but restrictions apply to the availability of these data, which were used under license for the current study, and so are not publicly available. Data are however available from the authors upon reasonable request and with permission of the Chief Investigator of the Transition project; Professor Allan Colver (allan.colver@newcastle.ac.uk).

## Abbreviations

NICE: National Institute for Care and Excellence
NHS: National Health Service
PSS: Personal and Social Services
IQWiG: Institut für Qualität und Wirtschaftlichkeit im Gesundheitswesen.
NIHR: National Institute for Health Research
ASD: Autistic Spectrum Disorder
CP: Cerebral Palsy
IMD: Index of Multiple Deprivation
YP: Young Person
HTA: Health Technology Assessment
