# Supplementary material for: Exploration of the costs of accessing health services: data from a longitudinal study of young people in transition from paediatric to adult services
Source: BMC Health Serv Res. 2021 Mar 21;21:263. doi: 10.1186/s12913-021-06280-z (PMC7981799; doi:10.1186/s12913-021-06280-z)
Supplement: Supplementary file 1 — Additional file 1. [file 12913_2021_6280_MOESM1_ESM.docx]

**Supplementary File 1** Comparison of sample characteristics between participants with complete and incomplete data

|  | Paired Analysis | Participants with Missing Total Costs at either T1 or T2 | Participants with Missing Total Costs at T1 | Participants with Missing Total Costs at T2 |
| --- | --- | --- | --- | --- |
|  | Mean (SD) | | | |
| n (% of Total Sample, N=299) | 221 (74) | 78 (26) | 12 (4) | 66 (22) |
| Age | 20.04 (1.30) | 20.06 (1.35) | 21.08 (1.16) | 19.88 (1.31) |
| %F | 43 | 40 | 58 | 36 |
| %Diabetes | 45 | 37 | 8 | 42 |
| %CP | 25 | 32 | 25 | 33 |
| %ASD | 30 | 31 | 66 | 24 |
| %Transferred at T1 | 29 | 35 | 50 | 32 |
| %Transferred at T2 | 56 | 64 | 67 | 64 |
| % IMD Quintile 1 | 16 | 14 | 8 | 15 |
| % IMD Quintile 2 | 18 | 21 | 8 | 23 |
| % IMD Quintile 3 | 22 | 14 | - | 17 |
| % IMD Quintile 4 | 22 | 21 | - | 25 |
| % IMD Quintile 5 | 22 | 15 | - | 19 |
| Total Cost at Time Point 1 | 246.73 (407.39) | 199.75 (182.64) | N/A | 199.75 (182.64) |
| Total Cost at Time Point 2 | 196.06 (237.05) | 232.00 (504.45) | 232.00 (504.45) | N/A |
