# Supplementary material for: Exploration of the costs of accessing health services: data from a longitudinal study of young people in transition from paediatric to adult services
Source: BMC Health Serv Res. 2021 Mar 21;21:263. doi: 10.1186/s12913-021-06280-z (PMC7981799; doi:10.1186/s12913-021-06280-z)
Supplement: Supplementary file 2 — Additional file 2. [file 12913_2021_6280_MOESM2_ESM.docx]

*Supplementary File 2 – Visit-level characteristics*

| Visit characteristics | Time point 1 | | | Time point 2 | | | |
| --- | --- | --- | --- | --- | --- | --- | --- |
|  | Diabetes | ASD | Cerebral Palsy | | Diabetes | ASD | Cerebral Palsy |
| n (total number of visits) | 243 | 136 | 191 | | 278 | 134 | 168 |
| Median time (minutes) per visit* | 120 | 90 | 100 | | 90 | 75 | 90 |
| Median miles travelled | 11 | 7 | 16.5 | | 8 | 7 | 17 |
| % Primary Care | 21 | 15 | 12 | | 27 | 25 | 12 |
| % Hospital | 52 | 20 | 64 | | 61 | 24 | 75 |
| % Community | 9 | 39 | 12 | | 6 | 33 | 7 |
| % CAMHS** | 0 | 24 | 4 | | 1 | 13 | 2 |
| %Unspecified | 18 | 2 | 9 | | 6 | 3 | 2 |

*Includes both visit and travel time

**Child and Adolescent Mental Health Services
